# High triglyceride glucose-body mass index correlates with prehypertension and hypertension in east Asian populations: A population-based retrospective study

**DOI:** 10.3389/fcvm.2023.1139842

**Published:** 2023-04-25

**Authors:** Lu Chen, Linfeng He, Wenbin Zheng, Qiuying Liu, Yifan Ren, Wen Kong, Tianshu Zeng

**Affiliations:** ^1^ Department of Endocrinology, Union Hospital, Tongji Medical College, Huazhong University of Science and Technology, Wuhan, China; ^2^ Hubei Provincial Clinical Research Center for Diabetes and Metabolic Disorders, Huazhong University of Science and Technology, Wuhan, China; ^3^ Hubei Key Laboratory of Metabolic Abnormalities and Vascular Aging, Huazhong University of Science and Technology, Wuhan, China

**Keywords:** triglyceride glucose-body mass index, prehypertension, hypertension, Chinese, Japanese

## Abstract

**Background:**

There is compelling evidence for an association between triglyceride glucose-body mass index (TyG-BMI) and cardiovascular disease (CVD). However, data on the relationship between TyG-BMI and prehypertension (pre-HTN) or hypertension (HTN) remains scant. The aim of this study was to characterize the association between TyG-BMI and pre-HTN or HTN risk, and to assess the ability of TyG-BMI in predicting pre-HTN and HTN in Chinese and Japanese populations.

**Methods:**

A total of 214,493 participants were included in this study. The participants were divided into 5 groups based on quintiles of TyG-BMI index at baseline (Q1, Q2, Q3 Q4 and Q5). Logistic regression analysis was then employed to assess the relationship between TyG-BMI quintiles and pre-HTN or HTN. Results were presented as odds ratios (ORs) and 95% confidence intervals (CIs).

**Results:**

Our restricted cubic spline analysis showed that TyG-BMI was linearly correlated with both pre-HTN and HTN. Multivariate logistic regression analysis indicated that TyG-BMI was independently correlated with pre-HTN [ORs and 95% CIs were 1.011 (1.011–1.012), 1.021 (1.02–1.023), 1.012 (1.012–1.012), respectively] and HTN [ORs and 95% CIs were 1.021 (1.02–1.021), 1.031 (1.028–1.033), 1.021 (1.02–1.021), respectively] in Chinese or Japanese individuals or both groups after adjusting for all variates. In addition, subgroup analyses showed that the relationship between TyG-BMI and pre-HTN or HTN was independent of age, sex, BMI, country, smoking and drinking status. Across all study populations, the areas under the TyG-BMI curve predicting pre-HTN and HTN were 0.667 and 0.762, respectively, resulting in cut-off values of 189.7 and 193.7, respectively.

**Conclusion:**

Our analyses showed that TyG-BMI was independently correlated with both pre-HTN and HTN. Besides, TyG-BMI showed superior predictive power in predicting pre-HTN and HTN compared to TyG or BMI alone.

## 1. Introduction

Hypertension is perceived as a preventable condition associated with an increased risk of cardiovascular disease (CVD) such as ischemic heart disease and stroke (1), which is a major contributor to all-cause mortality (2). Prehypertension is defined as a diastolic blood pressure (DBP) of 120–139 mmHg and/or systolic blood pressure (SBP) of 80–89 mmHg, which was first described in the JNC-7 report (3). In addition, data from the Framingham Heart Study suggest that older people with pre-HTN are more likely to progress to HTN within 4 years (4). It is estimated that one in three healthy individuals suffers from pre-HTN worldwide (5), and this rate will increase in future, with the number of adults diagnosed with HTN expected to reach 1.56 billion by 2025 (6). Therefore, there is an urgent need for early screening for pre-HTN and HTN in high-risk populations, along with development of effective interventions.

As a pathophysiological basis of various cardiometabolic diseases, insulin resistance (IR) has gained significant research attention in recent years (7–10). For instance, recent data has demonstrated an association between IR and the risk of pre-HTN (11–13) and HTN (14). Although hyperinsulinemic-euglycemic clamp (HEC) remains the gold standard for evaluating IR (15), it is not suitable for routine physical examinations and large population-based epidemiologic studies due to its invasiveness, required resources and time. Thus, more accessible and reproducible alternatives to IR are warranted in resource-limited primary care settings.

More recent data suggest that TyG-BMI, an emerging index calculated from triglycerides, fasting blood glucose and body mass index, has a great potential to be a surrogate for IR (16). Indeed, compelling evidence suggests a reliable relationship between TyG-BMI and nonalcoholic fatty disease (17), metabolic syndrome (18), diabetes (19) and ischemic stroke (20). However, data on the relationship between TyG-BMI and pre-HTN or HTN remains scant. Although both China and Japan are in East Asia, China is a developing country while Japan is a developed country. However, both countries have very low rates of blood pressure control. Data show that there are approximately 41.3% individuals with pre-HTN and 23.3% with HTN in China (21). On the other hand, 43 million people in Japan suffer from HTN, of which 31 million are poorly controlled. Worryingly, pre-HTN and HTN are responsible for approximately 50% of cardiovascular disease mortality (22). In this study, we aimed to characterize the relationship between TyG-BMI and the risk of pre-HTN or HTN, and determine its predictive power for reducing the incidence of early cardiovascular disease in Chinese and Japanese populations.

## 2. Materials and methods

### 2.1. Study population

**Figure 1:**
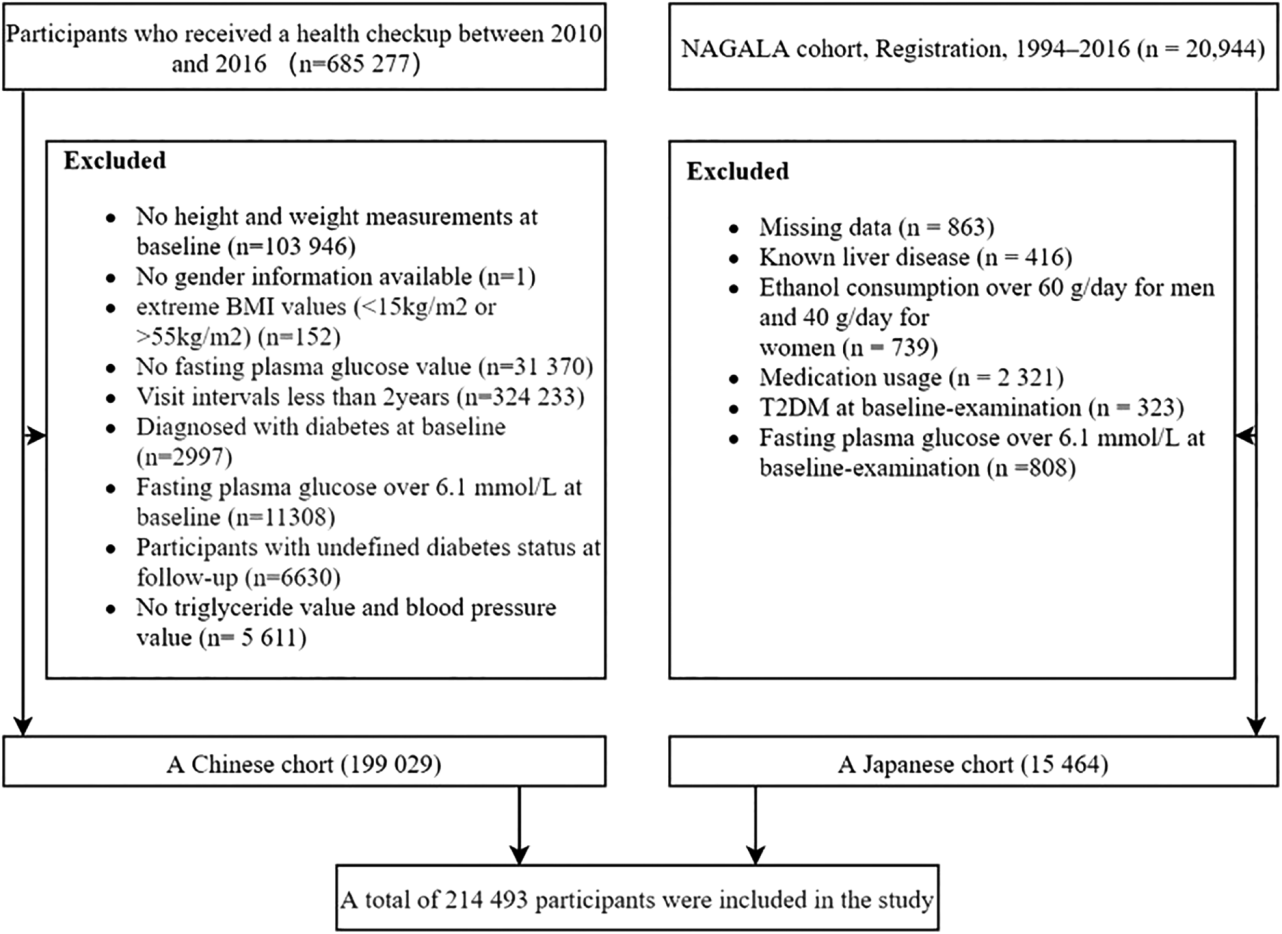
Flowcharts for study subjects.

This study included and analyzed two cohort studies: the Rich Health Care Group study and the NAGALA cohort study. The Rich Health Care Group study is a cohort study that recruited 685,277 participants who underwent health screening between 2010 and 2016 in 11 cities located in China (23). As shown in Figure 1, after applying our inclusion and exclusion criteria, we included and analyzed 199,029 subjects. The NAGALA cohort is a study that includes participants who have attended health checkups since Murakami Memorial Hospital was officially established in 1994 (24). In our study, we extracted data from 15,464 subjects in the NAGALA database from 1994 to 2016 (Figure 1**)**.The Ethics Committees had previously authorized the original project. We did not apply for ethical approval again, as our study was considered a *post hoc* analysis. All subjects provided informed consent in previous studies (24, 25). Our study complies with the Declaration of Helsinki.

### 2.2. Data collection and measurement

A standardized questionnaire which was completed by all subjects contained data on demographic characteristics, medical history, physical examination results and lifestyle information, which included age, sex, height, weight as well as the status of blood pressure, cigarette and alcohol consumption. Height, weight and blood pressure were measured by trained staff. Height was measured to the nearest 0.1 cm without shoes using a portable stadiometer, and weight was measured to the nearest 0.1 kg while participants were wearing lightweight clothing. BMI (kg/m^2^) was calculated as weight (kilograms) divided by height (meters) squared. All participants were required to rest in a quiet environment for at least 5 min before blood pressure measurement. Blood pressure was measured by standard mercury sphygmomanometers. Blood sample collection was conducted after fasting for at least 10 h. Fasting blood glucose (FPG), triglyceride (TG), total cholesterol (TC), and alanine aminotransferase (ALT), were measured by the uniform automated analyzer.

### 2.3. Definition and calculation

The participants were divided into three categories according to the criteria for hypertension described in the 2018 Chinese guidelines (26). BMI = weight (kg)/height (m)^2^; TyG = Ln [fasting TG (mg/dl)*FPG (mg/dl)/2] (27); TyG-BMI = TyG × BMI (16);

### 2.4. Statistical analysis

Stata/MP 17.0, EmpowerStats and R language software were used for data analysis. Continuous variables were grouped into normal and skewed variables. The normal variables were presented as mean ± standard deviation while the skewed variables were presented as median and interquartile range (IQR). Differences among continuous variables were tested by one-way ANOVA. Categorical variables were expressed as numbers (percentages) and then analyzed by chi-square tests. The relationship between TyG-BMI and BP or pre-HTN and HTN was analyzed by Spearman's correlation analysis and restricted cubic spline, respectively. In addition, we employed analytical models to examine the relationship between TyG-BMI and pre-HTN or HTN in the logistic regression analyses, including crude model (unadjusted), adjusted model I (adjusted for age and sex) and adjusted model II (adjusted model I + adjusted for smoking status, alcohol consumption, TC and ALT levels). Correlations with gender, age, BMI, country, smoking status and alcohol consumption were further tested. A *P *< 0.05 was defined as statistically significant.

## 3. Results

### 3.1. Study population baseline characteristics

This study recruited 214,493 participants (mean age: 41.95 ± 12.28, female/male: 98,041/116,452) including 199,029 Chinese and 15,464 Japanese. Of all participants, 27,168 had hypertension, 48,396 had prehypertension while 138,929 had normotension (Table 1). Age, proportion of males, BMI, SBP, DBP, FPG, TC, TG and ALT were highest in the hypertensive group. Interestingly, prehypertensives were more likely to drink alcohol and smoke compared to the other groups.

**Table 1:** Baseline Characteristics of study population.

	Total	Normal	Prehypertension	Hypertension	*P* value
*N*	214,493	138,929	48,396	27,168	
Age (years)	41.95 ± 12.28	39.69 ± 10.57	43.30 ± 12.74	51.05 ± 14.73	< 0.001^a^
Sex					< 0.001^c^
Woman	98,041 (45.7)	73,945 (53.2)	15,736 (32.5)	8,360 (30.8)	
Man	116,452 (54.3)	64,984 (46.8)	32,660 (67.5)	18,808 (69.2)	
Country					< 0.001^c^
Chinese	199,029 (92.8)	127,008 (91.4)	45,817 (94.7)	26,204 (96.5)	
Japanese	15,464 (7.2)	11,921 (8.5)	2,579 (5.3)	964 (3.5)	
BMI (kg/m^2^)	23.09 ± 3.31	22.35 ± 3.01	24.03 ± 3.31	25.19 ± 3.41	< 0.001^a^
SBP (mmHg)	118.37 ± 16.13	110.01 ± 10.19	127.29 ± 8.37	145.23 ± 13.35	< 0.001^a^
DBP (mmHg)	73.79 ± 10.74	68.06 ± 6.60	81.15 ± 5.68	90.03 ± 10.08	< 0.001^a^
FPG (mg/dl)	87.93 ± 9.86	86.93 ± 9.66	89.25 ± 9.88	90.74 ± 9.97	< 0.001^a^
TC (mg/dl)	182.94 ± 34.76	179.53 ± 33.64	186.76 ± 35.44	193.60 ± 36.29	< 0.001^a^
TG (mg/dl)	91.26 (62.91,138.20)	81.51 (58.48,119.60)	107.20 (73.54,159.50)	125.80 (87.71,185.20)	< 0.001^b^
ALT (U/l)	18.00 (12.90, 26.90)	16.10 (12.00, 24.00)	20.60 (14.60, 31.10)	22.00 (16.00, 33.32)	< 0.001^b^
TyG-BMI	193.32 ± 36.75	184.27 ± 33.08	204.97 ± 36.49	218.88 ± 37.17	< 0.001^a^
Smoking					< 0.001^c^
No	54,579 (25.4)	36,980 (26.6)	12,203 (25.2)	5,396 (19.9)	
Yes	16,978 (7.9)	9,818 (7.1)	4,799 (9.9)	2,361 (8.7)	
Unkonwn	142,936 (66.6)	92,131 (66.3)	31,394 (64.9)	19,411 (71.4)	
Drinking					< 0.001^c^
No	55,539 (25.9)	36,727 (26.4)	12,881 (26.6)	5,931 (21.8)	
Yes	16,018 (7.5)	10,071 (7.2)	4,121 (8.5)	1,826 (6.7)	
Unkonwn	142,936 (66.6)	92,131 (66.3)	31,394 (64.9)	19,411 (71.4)	

Continuous variables are shown as mean ± standard deviation if normally distributed or median (interquartile range) if non-normally distributed. Categorical variables are expressed as numbers and percentages of subjects.

^a^ *P *< 0.001 means differences among variables are tested by one-way analysis of variance.

^b^ *P *< 0.001 means differences among variables are tested by rank sum test.

^c^ *P *< 0.001 means differences among variables are tested by the chi-square test.

BMI body mass index; SBP systolic blood pressure; DBP diastolic blood pressure; FPG fasting plasma glucose; TC total cholesterol; TG triglyceride; HDL high-density lipoprotein; ALT alanine aminotransferase.

### 3.2. Logistic regression analyses of TyG-BMI quintiles and prehypertension and hypertension

**Figure 2:**
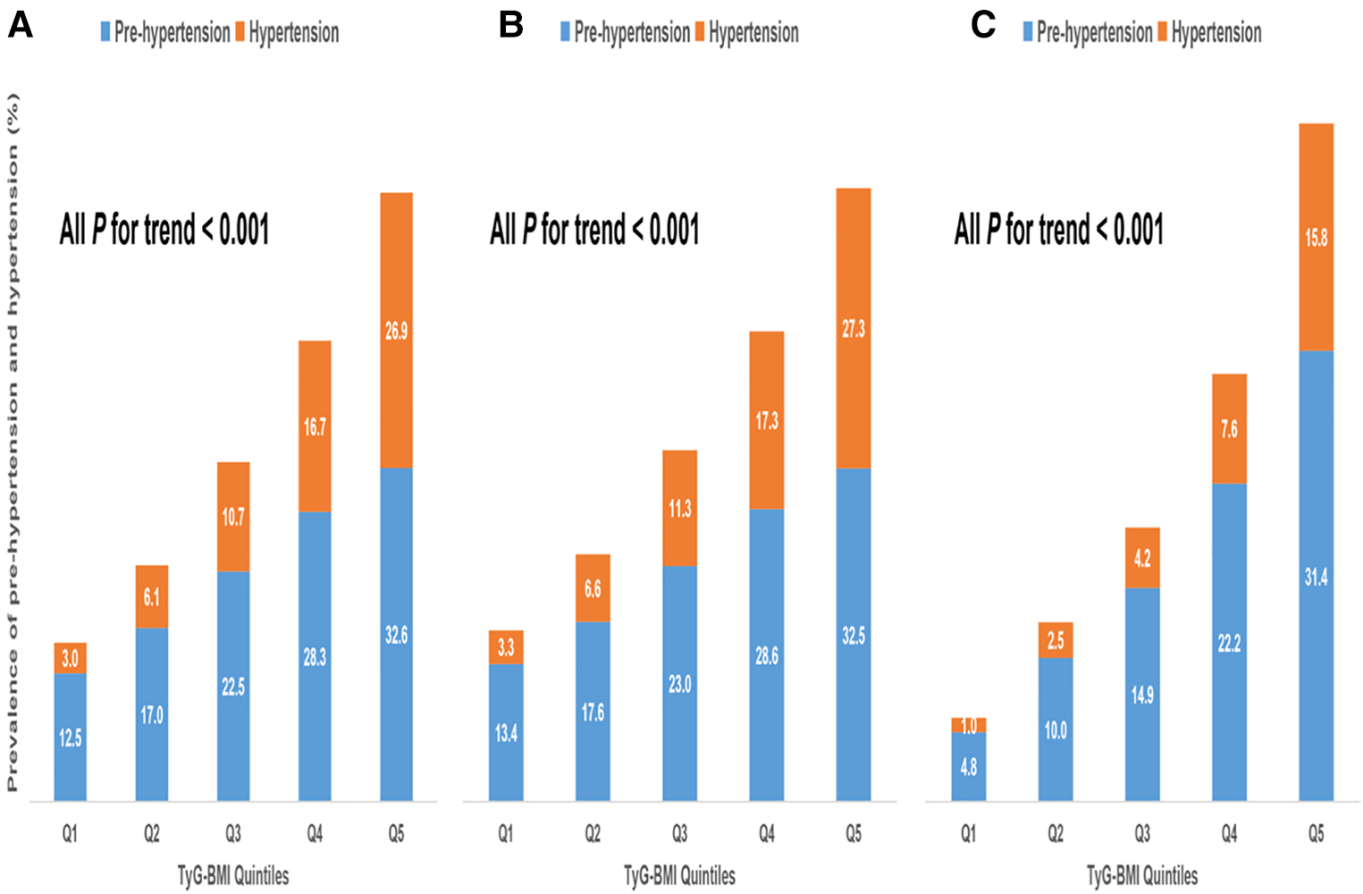
Prevalence of prehypertension and hypertension according to TyG-BMI group. (**A**). All populations (**B**). Chinese (**C**). Japanese.

Our data showed that age, BMI, SBP, DBP, FPG, TC, TG, ALT, TyG and TyG-BMI were all positively correlated with pre-HTN and HTN (Supplementary Table S1). Men and those with high drinking and smoking behaviors had a higher likelihood of developing pre-HTN and HTN. Comparison of the two geographical populations demonstrated that Chinese were more prone to pre-HTN and HTN than Japanese (Supplementary Table S1). In addition, there was a linear relationship between TyG-BMI and SBP or DBP (Supplementary Fig. S1) and between TyG-BMI and pre-HTN or HTN (Supplementary Fig. S2). Analysis of TyG-BMI quintiles showed that the prevalence of pre-HTN and HTN increased with increasing TyG-BMI quintiles in either Chinese or Japanese or both (Figure 2). Odds ratios (ORs) for TyG-BMI were positively correlated with pre-HTN or HTN regardless of whether confounders were adjusted (Table 2 and Table 3). In the adjusted model II with the quintile1 as a reference in both the Chinese and Japanese populations, the ORs and 95% CIs for pre-HTN were 1.012 (1.012–1.012), 1.011 (1.011–1.012) and 1.021 (1.02–1.023), whereas the ORs and 95% CIs for HTN were 1.021 (1.02–1.021), 1.021 (1.02–1.021) and 1.031 (1.028–1.033), respectively.

**Table 2:** Logistic regression analysis of the relationship between TyG-BMI and prehypertension.

	Crude model		Adjusted model I		Adjusted model II	
	OR (95% CI)	*P* value	OR (95% CI)	P value	OR (95% CI)	*P* value
**All populations**						
TyG-BMI	1.017 (1.016, 1.017)	<0.001	1.013 (1.013, 1.013)	<0.001	1.012 (1.012, 1.012)	<0.001
TyG-BMI Quintiles						
Q1	1 (Ref)	<0.001	1 (Ref)	<0.001	1 (Ref)	<0.001
Q2	1.487 (1.431, 1.545)	<0.001	1.296 (1.247, 1.347)	<0.001	1.256 (1.208, 1.306)	<0.001
Q3	2.269 (2.187, 2.354)	<0.001	1.74 (1.675, 1.808)	<0.001	1.646 (1.584, 1.71)	<0.001
Q4	3.469 (3.346, 3.596)	<0.001	2.415 (2.325, 2.509)	<0.001	2.211 (2.127, 2.299)	<0.001
Q5	5.418 (5.224, 5.618)	<0.001	3.608 (3.471, 3.75)	<0.001	3.163 (3.037, 3.295)	<0.001
*P* for trend	< 0.001		< 0.001		< 0.001	
**Chinese**						
TyG-BMI	1.016 (1.016, 1.016)	<0.001	1.012 (1.012, 1.013)	<0.001	1.011 (1.011,1.012)	<0.001
TyG-BMI Quintiles						
Q1	1 (Ref)		1 (Ref)		1 (Ref)	
Q2	1.434 (1.38, 1.491)	<0.001	1.243 (1.195, 1.293)	<0.001	1.224 (1.176,1.273)	<0.001
Q3	2.169 (2.09, 2.252)	<0.001	1.644 (1.581, 1.709)	<0.001	1.592 (1.531,1.656)	<0.001
Q4	3.271 (3.153, 3.393)	<0.001	2.237 (2.152, 2.326)	<0.001	2.121 (2.038,2.207)	<0.001
Q5	5.024 (4.841, 5.213)	<0.001	3.283 (3.156, 3.416)	<0.001	3.009 (2.887,3.137)	<0.001
*P* for trend	< 0.001		< 0.001		< 0.001	
**Japanese**						
TyG-BMI	1.025 (1.023, 1.026)	<0.001	1.021 (1.02, 1.023)	<0.001	1.021 (1.02, 1.023)	<0.001
TyG-BMI Quintiles						
Q1	1 (Ref)		1 (Ref)		1 (Ref)	
Q2	2.224 (1.818, 2.721)	<0.001	1.816 (1.48, 2.228)	<0.001	1.781 (1.451,2.187)	<0.001
Q3	3.578 (2.954, 4.335)	<0.001	2.513 (2.062, 3.062)	<0.001	2.424 (1.987, 2.959)	<0.001
Q4	6.134 (5.097, 7.382)	<0.001	3.879 (3.194, 4.71)	<0.001	3.696 (3.034, 4.503)	<0.001
Q5	11.569 (9.641,13.883)	<0.001	7.367 (6.08, 8.925)	<0.001	6.666 (5.452, 8.149)	<0.001
*P* for trend	< 0.001		< 0.001		< 0.001	

Crude model: unadjusted.

Adjusted model I: adjusted for age and sex.

Adjusted model II: adjusted for Adjusted model II + TC + ALT + smoking + drinking.

BMI body mass index; TC total cholesterol; TyG triglyceride glucose index; ALT alanine aminotransferase; OR odds ratio; CI confidence interval.

**Table 3:** Logistic regression analysis of the relationship between TyG-BMI and hypertension.

	Crude model		Adjusted model I		Adjusted model II	
	OR (95%CI)	*P* value	OR (95%CI)	*P* value	OR (95%CI)	*P* value
**All populations**						
TyG-BMI	1.026 (1.026, 1.027)	<0.001	1.022 (1.022, 1.023)	<0.001	1.021 (1.02, 1.021)	< 0.001
TyG-BMI Quintiles						
Q1	1 (Ref)	<0.001	1 (Ref)	< 0.001	1 (Ref)	< 0.001
Q2	2.251 (2.102, 2.41)	<0.001	1.695 (1.579, 1.819)	< 0.001	1.615 (1.504, 1.733)	< 0.001
Q3	4.512 (4.233, 4.81)	<0.001	2.719 (2.544, 2.906)	< 0.001	2.5 (2.338, 2.673)	< 0.001
Q4	8.574 (8.061, 9.119)	<0.001	4.426 (4.149, 4.723)	< 0.001	3.891 (3.643, 4.156)	< 0.001
Q5	18.723 (17.624, 19.89)	<0.001	9.721 (9.12, 10.361)	< 0.001	7.985 (7.475, 8.529)	< 0.001
*P* for trend	<0.001		<0.001		<0.001	
**Chinese**						
TyG-BMI	1.026 (1.026, 1.026)	<0.001	1.021 (1.021, 1.022)	<0.001	1.021 (1.02, 1.021)	<0.001
TyG-BMI Quintiles						
Q1	1 (Ref)		1 (Ref)		1 (Ref)	
Q2	2.203 (2.058, 2.359)	<0.001	1.627 (1.516, 1.746)	<0.001	1.591 (1.482, 1.708)	<0.001
Q3	4.362 (4.093, 4.649)	<0.001	2.532 (2.369, 2.707)	<0.001	2.423 (2.265, 2.591)	<0.001
Q4	8.117 (7.633, 8.632)	<0.001	4.022 (3.768, 4.292)	<0.001	3.735 (3.497, 3.989)	<0.001
Q5	17.279 (16.266,18.354)	<0.001	8.619 (8.084, 9.189)	<0.001	7.58 (7.097, 8.097)	<0.001
*P* for trend	<0.001		<0.001		<0.001	
**Japanese**						
TyG-BMI	1.032 (1.03, 1.034)	<0.001	1.031 (1.028, 1.033)	<0.001	1.031 (1.028, 1.033)	<0.001
TyG-BMI Quintiles						
Q1	1 (Ref)		1 (Ref)		1 (Ref)	
Q2	2.673 (1.756, 4.069)	<0.001	2.089 (1.368, 3.19)	0.0007	2.02 (1.321, 3.088)	0.0012
Q3	4.883 (3.288, 7.251)	<0.001	3.175 (2.123, 4.749)	<0.001	2.956 (1.972, 4.432)	<0.001
Q4	10.211 (6.993, 14.91)	<0.001	6.004 (4.067, 8.863)	<0.001	5.603 (3.781, 8.302)	<0.001
Q5	28.221 (19.532,40.775)	<0.001	17.045 (11.657,24.923)	<0.001	14.89 (10.086, 21.983)	<0.001
*P* for trend	<0.001		<0.001		<0.001	

Crude model: unadjusted.

Adjusted model I: adjusted for age and sex.

Adjusted model II: adjusted for Adjusted model II + TC + ALT + smoking + drinking.

BMI body mass index; TC total cholesterol; TyG triglyceride glucose index; ALT alanine aminotransferase; OR odds ratio; CI confidence interval.

### 3.3. Association between TyG-BMI and pre-HTN or HTN in subgroups

To assess the impact of TyG-BMI on pre-HTN or HTN, we explored associations across age, gender, country, BMI, smoking and drinking status. Our analyses revealed significant differences in TyG-BMI-related pre-HTN and HTN risk across phenotypes. As shown in Supplementary Table S2, group-specific TyG-BMI was a stronger predictor of pre-HTN and HTN. We determined the ORs for each subgroup. In the age stratification, ORs for pre-HTN (<45 years: 1.012 vs. 45–60 years: 1.013 vs. > 60 years: 1.01; P-interaction < 0.001), ORs for HTN (<45 years: 1.015 vs. 45–60 years: 1.017 vs. > 60 years: 1.015; P-interaction < 0.001). In the sex stratification, ORs for pre-HTN (women: 1.013 vs. men: 1.011; P-interaction <0.001), ORs for HTN (women: 1.024 vs. men: 1.011; P-interaction <0.001). In the country stratification, ORs for pre-HTN (Japanese: 1.021 vs. Chinese: 1.011; P-interaction <0.001), ORs for HTN (Japanese: 1.022 vs. Chinese: 1.015; P-interaction <0.001). In the BMI stratification, ORs for pre-HTN (1.013 in BMI < 18.5 kg/m^2^ group vs. 1.013 in 18.5 kg/m^2^ ≤ BMI < 24 kg/m^2^ group vs. 1.01 in 24 kg/m^2^ ≤ BMI < 28 kg/m^2^ group vs. 1.008 in BMI ≥ 28 kg/m^2^ group; P-interaction < 0.001); ORs for HTN (1.032 vs. 1.022 vs. 1.014 vs. 1.009 for the corresponding group, respectively; P-interaction < 0.001).

### 3.4. Prediction of TyG-BMI in the pre-HTN or HTN

**Figure 3:**
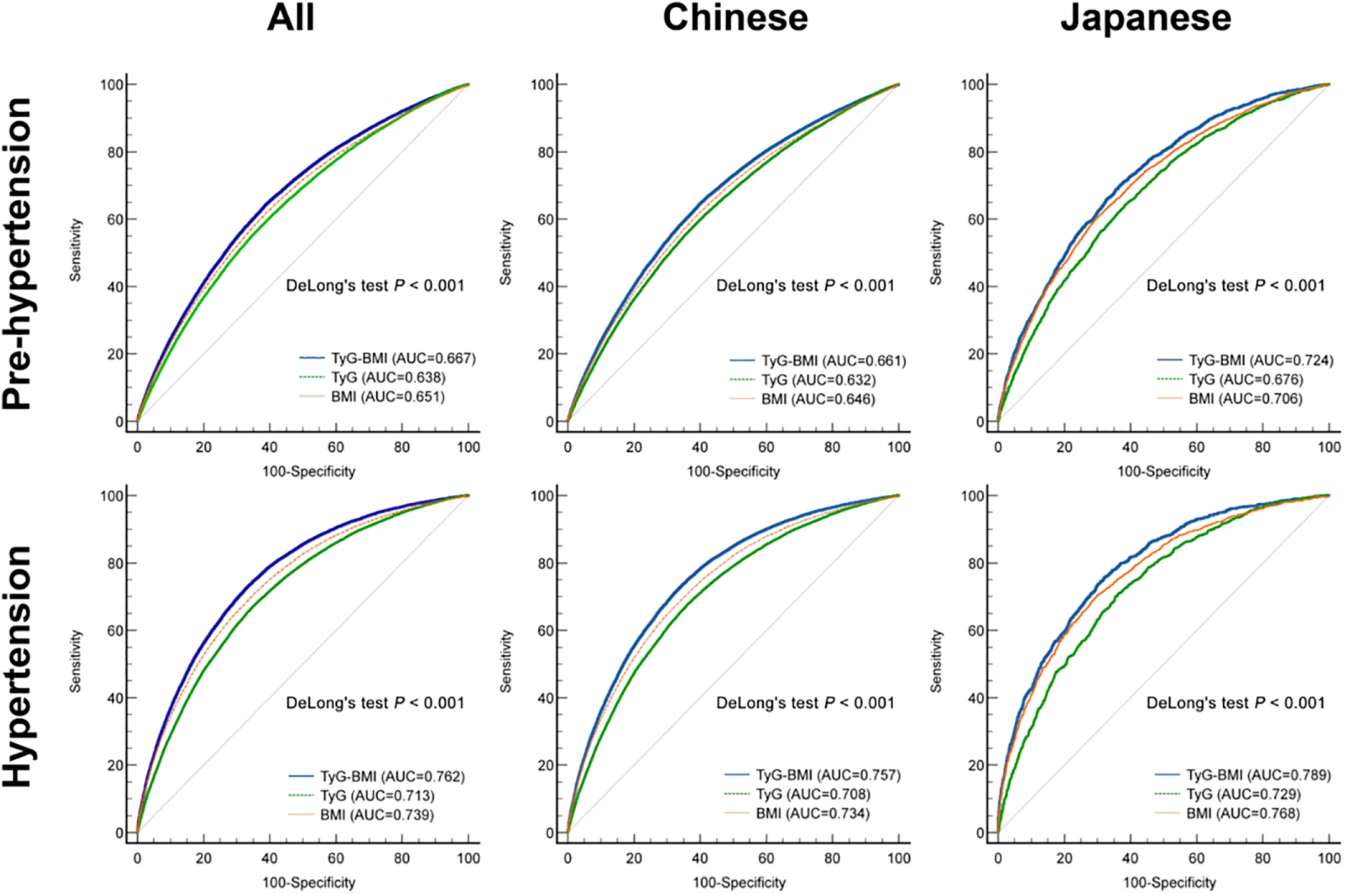
ROC curve analysis of TyG-BMI, TyG and BMI.

To characterize the predictive ability of BMI, TyG and TyG-BMI for pre-HTN and HTN, we performed receiver operating characteristic (ROC) curve analysis for the Chinese and Japanese patients (Figure 3). Prediction of pre-HTN and HTN in Chinese and Japanese showed that TyG-BMI exhibited the highest discriminative performance in both populations (*P *< 0.001). Across all populations, the AUCs of TyG-BMI for pre-HTN and HTN were 0.667 (95% CI: 0.665–0.670) and 0.762 (95% CI: 0.760, 0.764), respectively. In addition, the corresponding optimal cut-off values for TyG-BMI were 189.7 and 193.7 (Supplementary Table S3).

## 4. Discussion

We performed a retrospective study to evaluate the association between TyG-BMI and the risk of pre-HTN or HTN. The findings showed that TyG-BMI was positively correlated with pre-HTN or HTN regardless of whether the covariates were adjusted in all populations. TyG-BMI was reliable in predicting pre-HTN and HTN, thus it can be an accessible, resource- and time-saving indicator for early identification and intervention in pre-HTN and HTN.

IR has long been regarded as a core pathophysiological marker for diverse cardiometabolic abnormalities such as CVD (28). Hence, successful screening for IR at an early stage before the development of unfavorable life-threatening complications, will reduce the burden on health outcomes and the strain on the global economy. Nevertheless, the most precise evaluation method of IR remains HEC, which is invasive and labour-intensive (29). Besides, it is sophisticated and impractical to conduct a HEC experiment in large-scale population-based studies. Therefore, a number of simple, inexpensive and reproducible alternative parameters have emerged, which have been shown to replace HEC for IR. These parameters include the ratios of triglycerides and specific components of cholesterol, TyG and TyG-related parameters, metabolic score for insulin resistance (METS-IR), visceral adiposity index (VAI) and lipid accumulation product (LAP), whose association with IR has been explored (16, 30, 31) and compared in the prediction of some specific diseases (32–36).

Intriguingly, Er et al. (16) found that TyG-BMI is an optimum index substitute for IR in nondiabetic Taiwanese people compared to some indices related to lipids, adipokines and visceral adiposity. In addition, Li et al. (37) showed that Chinese visceral adiposity index (CVAI) had an optimal and superior discriminative ability in predicting pre-HTN or HTN compared to the VAI, which was based on 34,732 Chinese participants. On the other hand, Han et al. (38) observed that METS-IR was strongly associated with pre-HTN and HTN in 15,453 Japanese individuals with normal glucose tolerance. In recent years, TyG-BMI has been shown to have a higher value in predicting HTN prevalence compared to METS-IR in 117,056 Chinese participants (32).

Our study aimed to explore the role of TyG-BMI in predicting pre-HTN and HTN in 214,493 subjects from China and Japan. In addition, our findings suggest that middle-aged, nonobese, female and Japanese individuals have a greater ability of TyG-BMI in predicting pre-HTN and HTN. This may be due to several factors; (1) the accelerated pace of modern life and the rapidly increasing ageing population, which further exacerbates the social burden on middle-aged people (39). (2) Asians gain more body adiposity at lower BMI levels (40, 41) and are more likely to accumulate visceral fat (41, 42), which is more strongly associated with pre-HTN and HTN than subcutaneous fat. BMI alone does not reflect adiposity (43). (3) Sexual dimorphism between TyG-BMI and pre-HTN and HTN is due to gender differences in the renin-angiotensin system (RAS), sympathetic nervous activity (SNA), estrogens and androgens, ET-1 and immunoregulatory cytokines (44). (4) Consistent with the prevalence of obesity, the incidence of CVD in Japanese individuals is significantly lower than that in the Chinese individuals, which is robustly associated with their lifestyle habits, such as lower salt and red meat intake and higher physical activity. The observation that the relationship between TyG-BMI and pre-HTN and HTN is more obvious in Japanese individuals seems abnormal, but may be related to the ageing of the Japanese population. Future research is needed to determine the underlying mechanisms.

There is a rational assumption that TyG-BMI is an optimal substitute for IR as supported by various findings: (1) glucotoxicity and lipotoxicity are of paramount importance in the development of IR (45); (2) BMI can partially reflect the information on adipose tissue. Moreover, adipose tissues which are also deemed an endocrine organ, can secrete hormones, cytokines, chemokines and adipokines (i.e., leptin and adiponectin) with effects on glucose, lipid metabolism and signaling pathways mediating IR (46). Thus, IR is a pivotal factor underlying the mechanisms linking TyG-BMI and pre-HTN or HTN. High levels of insulin could activate RAS (47) and SNA (48), leading to the expansion of vascular smooth muscle and endothelial cells which further increases peripheral vascular resistance. In addition, IR promotes the absorption of salt. Moreover, IR can accelerate atherosclerosis by attenuating eNOS activation and damaging endothelial function, resulting in systemic vasoconstriction (49).

Although our study highlights important findings, the causal relationship between TyG-BMI and pre-HTN or HTN remains inconclusive as the study was a retrospective study. Secondly, only one race was involved in our study, and therefore the conclusions cannot be generalized into other ethnic groups. Thirdly, we did not take diet and exercise habits, family history, economic status and occupational stress into account, which could lead to confounding bias. Owing to high cost, we also did not assess fasting insulin or C-peptide levels, which partially reflect IR. Thus, more large-scale population-based randomized clinical trials or Mendelian randomization studies are needed to conclusively characterize the causal relationship between TyG-BMI and pre-HTN or HTN. Besides, our findings need to be validated in other races and countries due to genetic and habitual disparities. In addition, as life or work stress exerts adverse impacts on elevated blood pressure, this should be taken into account in future studies.

## 5. Conclusion

In summary, our data suggest that TyG-BMI is significantly correlated with pre-HTN and HTN, and exhibited excellent power in predicting the risk of pre-HTN and HTN in East Asian populations. Therefore, TyG-BMI is a promising tool and might be an optimal indicator for screening high-risk individuals susceptible to pre-HTN and HTN, and may be a novel target for the prevention and management of pre-HTN and HTN.

## Data Availability

The datasets presented in this study can be found in online repositories. The names of the repository/repositories and accession number(s) can be found below: The data used in this study can be obtained from the ‘DATADRYAD' database (www.Datadryad.org).
