# Supplementary material for: High triglyceride glucose-body mass index correlates with prehypertension and hypertension in east Asian populations: A population-based retrospective study
Source: Front Cardiovasc Med. 2023 Apr 25;10:1139842. doi: 10.3389/fcvm.2023.1139842 (PMC10166815; doi:10.3389/fcvm.2023.1139842)
Supplement: Supplementary file 1 [file Datasheet1.doc]

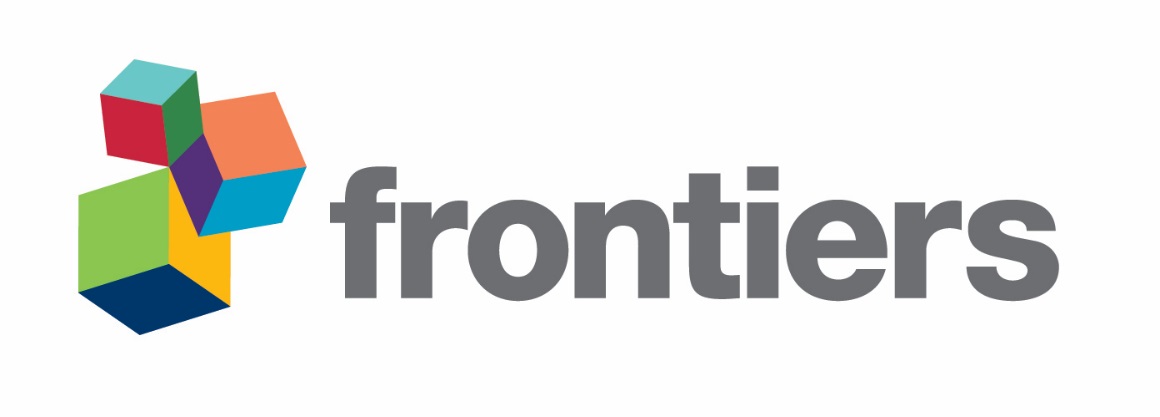


Supplementary Material

**Figure legends**

**Supplementary Fig. 1.**Scatterplot of TyG-BMI and blood pressure. A All populations, B Chinese, C Japanese.

**Supplementary Fig. 2.**Restricted cubic splines of 4 knots in prehypertension and hypertension.

| **Supplementary table 1** Univariate analysis of pre-hypertension and hypertension | | | | | |
| --- | --- | --- | --- | --- | --- |
|  | **Pre-hypertension** | |  | **Hypertension** | |
|  | **OR (95%CI)** | ***P*-value** |  | **OR (95%CI)** | ***P*-value** |
| Age | 1.027 (1.026, 1.028) | <0.001 |  | 1.072 (1.071, 1.074) | <0.001 |
| Sex |  |  |  |  |  |
| Woman | Reference |  |  | Reference |  |
| Man | 2.362 (2.311, 2.414) | <0.001 |  | 2.56 (2.49, 2.632) | <0.001 |
| Country |  |  |  |  |  |
| Chinese | Reference |  |  | Reference |  |
| Japanese | 0.6 (0.574, 0.627) | <0.001 |  | 0.392 (0.367, 0.419) | <0.001 |
| BMI | 1.182 (1.178, 1.186) | <0.001 |  | 1.304 (1.298, 1.309) | <0.001 |
| SBP | 1.22 (1.217, 1.222) | <0.001 |  | 1.469 (1.46, 1.479) | <0.001 |
| DBP | 1.469 (1.463, 1.474) | <0.001 |  | 1.53 (1.52, 1.539) | <0.001 |
| FPG | 1.025 (1.024, 1.026) | <0.001 |  | 1.043 (1.042, 1.045) | <0.001 |
| TC | 1.006 (1.006, 1.006) | <0.001 |  | 1.011 (1.011, 1.012) | <0.001 |
| TG | 1.005 (1.005, 1.005) | <0.001 |  | 1.007 (1.006, 1.007) | <0.001 |
| [ALT](../../../../D:/%25E5%25AE%2589%25E8%25A3%2585%25E7%25A8%258B%25E5%25BA%258F/Youdao/Dict/9.0.4.0/resultui/html/index.html#\\javascript:;) | 1.014 (1.014, 1.015) | <0.001 |  | 1.016 (1.016, 1.017) | <0.001 |
| TyG | 2.236 (2.197, 2.277) | <0.001 |  | 3.458 (3.38, 3.538) | <0.001 |
| TyG-BMI | 1.017 (1.016, 1.017) | <0.001 |  | 1.026 (1.026, 1.027) | <0.001 |
| Smoking |  |  |  |  |  |
| No | Reference |  |  | Reference |  |
| Yes | 1.481 (1.423, 1.542) | <0.001 |  | 1.648 (1.563, 1.738) | <0.001 |
| Unkonwn | 1.033 (1.008, 1.058) | 0.009 |  | 1.444 (1.398, 1.492) | <0.001 |
| Drinking |  |  |  |  |  |
| No | Reference |  |  | Reference |  |
| Yes | 1.167 (1.119, 1.216) | <0.001 |  | 1.123 (1.061, 1.188) | <0.001 |
| Unkonwn | 0.972 (0.949, 0.995) | 0.0176 |  | 1.305 (1.264, 1.346) | <0.001 |
| BMI body mass index; SBP systolic blood pressure; DBP diastolic blood pressure; FPG fasting plasma glucose; TC total cholesterol; TG triglyceride; HDL high-density lipoprotein; ALT Alanine aminotransferase; OR odds ratio, CI confidence interval | | | | | |

| **Supplementary table 2** Subgroup analysis of TyG-BMI and prehypertension, hypertension | | | | | |
| --- | --- | --- | --- | --- | --- |
| **Subgroup** | **Pre-hypertension** | |  | **Hypertension** | |
|  | **Adjusted OR (95%CI)** | ***P*_interaction_** |  | **Adjusted OR (95%CI)** | ***P*_interaction_** |
| **Age group** |  | <0.001 |  |  | <0.001 |
| <45 | 1.012 (1.012, 1.013) |  |  | 1.015 (1.013, 1.016) |  |
| 45-60 | 1.013 (1.012, 1.013) |  |  | 1.017 (1.015, 1.019) |  |
| ≥60 | 1.01 (1.009, 1.011) |  |  | 1.015 (1.012, 1.018) |  |
| **Sex** |  | <0.001 |  |  | <0.001 |
| Woman | 1.013 (1.013, 1.014) |  |  | 1.024 (1.022, 1.026) |  |
| Man | 1.011 (1.01, 1.011) |  |  | 1.011 (1.01, 1.013) |  |
| **Country** |  | <0.001 |  |  | <0.001 |
| Chinese | 1.011 (1.011, 1.012) |  |  | 1.015 (1.014, 1.017) |  |
| Japanese | 1.021 (1.02, 1.023) |  |  | 1.022 (1.016, 1.028) |  |
| **BMI group** |  | <0.001 |  |  | <0.001 |
| <18.5 | 1.013 (1.007, 1.019) |  |  | 1.032 (1.018, 1.047) |  |
| 18.5-24 | 1.013 (1.012, 1.014) |  |  | 1.022 (1.02, 1.025) |  |
| 24-28 | 1.01 (1.009, 1.011) |  |  | 1.014 (1.012, 1.015) |  |
| ≥28 | 1.008 (1.006, 1.01) |  |  | 1.009 (1.007, 1.012) |  |
| **Smoking** |  | <0.001 |  |  | 0.006 |
| No | 1.013 (1.013, 1.014) |  |  | 1.017 (1.014, 1.02) |  |
| Yes | 1.012 (1.01, 1.013) |  |  | 1.012 (1.009, 1.016) |  |
| Unkonwn | 1.012 (1.011, 1.012) |  |  | 1.015 (1.014, 1.017) |  |
| **Drinking** |  | 0.003 |  |  | 0.015 |
| No | 1.013 (1.012, 1.013) |  |  | 1.016 (1.014, 1.019) |  |
| Yes | 1.013 (1.012, 1.015) |  |  | 1.014 (1.01, 1.018) |  |
| Unkonwn | 1.012 (1.011, 1.012) |  |  | 1.015 (1.014, 1.017) |  |
| Adjusted for age, sex, TC, ALT, smoking and drinking BMI body mass index; TyG-BMI triglyceride glucose indexOR odds ratio; CI confidence interval | | | | | |

| **Supplementary table 3** Accuracy of the relationship between TyG-BMI and pre-hypertension, hypettension | | | | | | |
| --- | --- | --- | --- | --- | --- | --- |
|  | **Cut-off** | **AUC** | **95%CI** | **Sensitivity (%)** | **Specificity (%)** | **Youden index** |
| **Pre-hypertension** |  |  |  |  |  |  |
| **All** |  |  |  |  |  |  |
| TyG-BMI | 189.7 | 0.667 | 0.665, 0.670 | 64.50 | 60.97 | 0.255 |
| TyG | 8.3 | 0.638 | 0.635, 0.640 | 60.89 | 50.60 | 0.205 |
| BMI | 23.0 | 0.651 | 0.649, 0.653 | 61.80 | 61.14 | 0.229 |
| **Chinese** |  |  |  |  |  |  |
| TyG-BMI | 189.7 | 0.661 | 0.659, 0.663 | 64.87 | 59.77 | 0.246 |
| TyG | 8.4 | 0.632 | 0.630, 0.634 | 55.83 | 64.07 | 0.199 |
| BMI | 22.9 | 0.646 | 0.643, 0.648 | 62.07 | 60.04 | 0.221 |
| **Japanese** |  |  |  |  |  |  |
| TyG-BMI | 178.8 | 0.724 | 0.717, 0.731 | 69.83 | 63.36 | 0.332 |
| TyG | 8.1 | 0.676 | 0.668, 0.683 | 64.29 | 61.63 | 0.259 |
| BMI | 22.8 | 0.706 | 0.698, 0.713 | 60.18 | 70.52 | 0.307 |
| **Hypertension** |  |  |  |  |  |  |
| **All** |  |  |  |  |  |  |
| TyG-BMI | 193.7 | 0.762 | 0.760, 0.764 | 74.60 | 65.02 | 0.396 |
| TyG | 8.4 | 0.713 | 0.711, 0.715 | 67.03 | 65.08 | 0.321 |
| BMI | 23.3 | 0.739 | 0.737, 0.741 | 70.93 | 64.59 | 0.355 |
| **Chinese** |  |  |  |  |  |  |
| TyG-BMI | 196.3 | 0.757 | 0.754, 0.759 | 72.44 | 66.47 | 0.389 |
| TyG | 8.4 | 0.708 | 0.705, 0.710 | 67.55 | 63.93 | 0.315 |
| BMI | 23.6 | 0.734 | 0.732, 0.737 | 66.78 | 67.99 | 0.348 |
| **Japanese** |  |  |  |  |  |  |
| TyG-BMI | 183.0 | 0.789 | 0.782, 0.796 | 75.73 | 67.92 | 0.437 |
| TyG | 8.1 | 0.729 | 0.722, 0.737 | 70.02 | 64.63 | 0.347 |
| BMI | 22.8 | 0.768 | 0.761, 0.775 | 70.44 | 70.12 | 0.406 |
| BMI body mass index; TyG triglyceride glucose index | | | | | | |
