# Supplementary figures and images for: High triglyceride glucose-body mass index correlates with prehypertension and hypertension in east Asian populations: A population-based retrospective study
Source: Front Cardiovasc Med. 2023 Apr 25;10:1139842. doi: 10.3389/fcvm.2023.1139842 (PMC10166815; doi:10.3389/fcvm.2023.1139842)

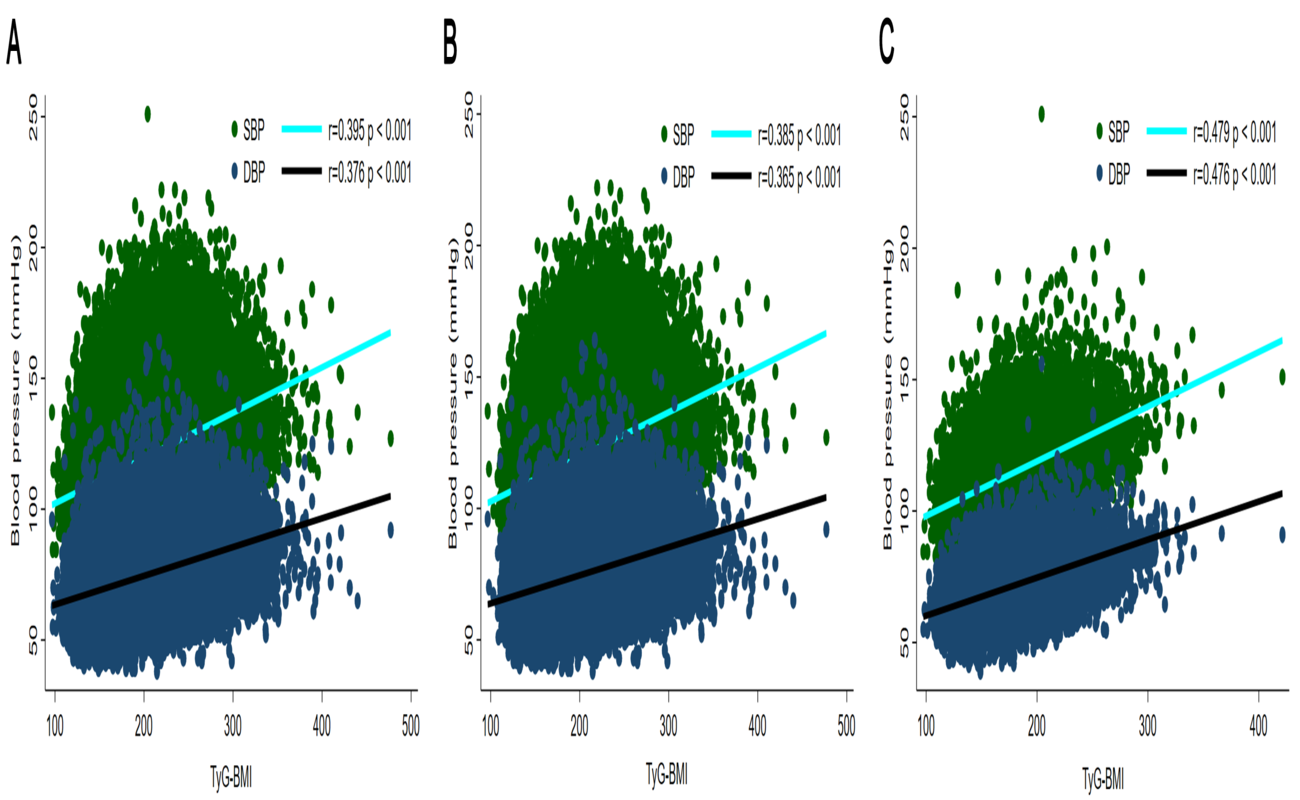

Supplement: Supplementary file 2 [file Image1.tiff]

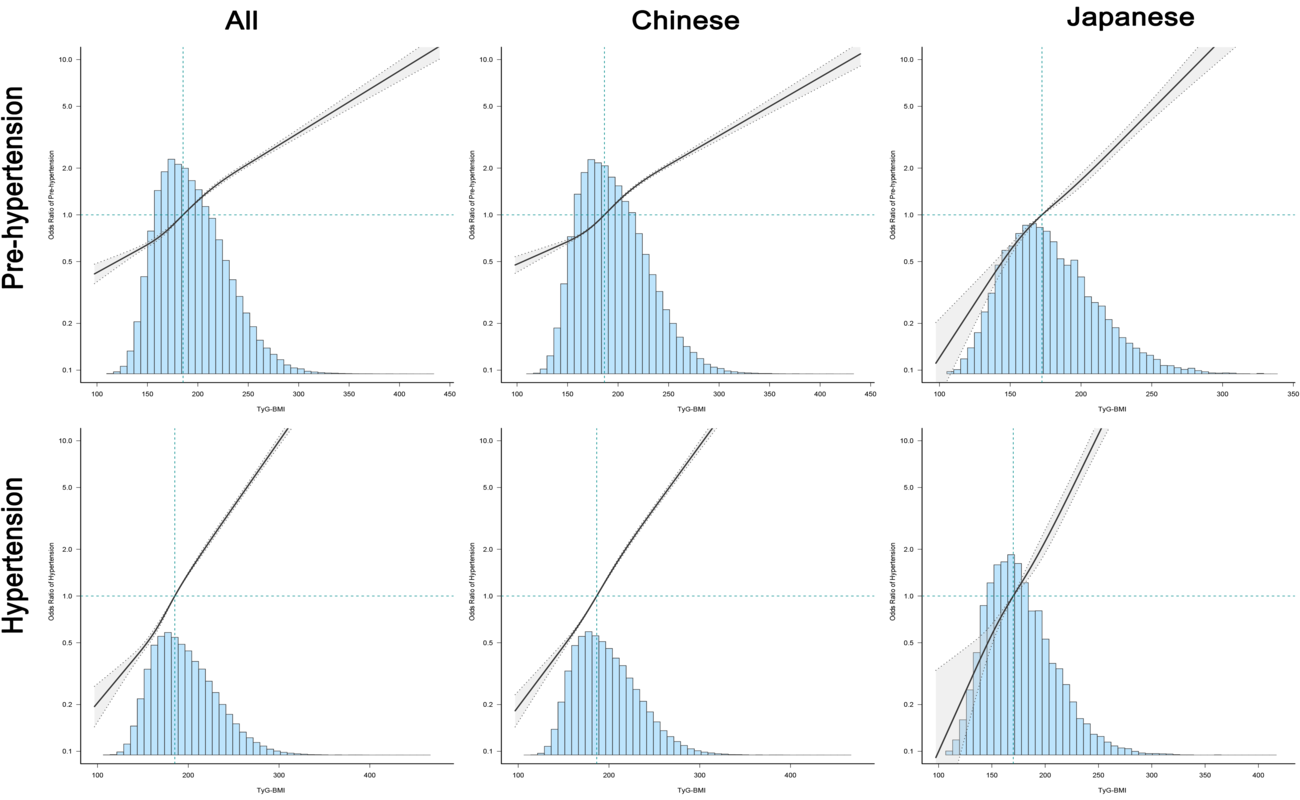

Supplement: Supplementary file 3 [file Image2.tiff]
